# Author Credit for Transdisciplinary Collaboration

**DOI:** 10.1371/journal.pone.0137968

**Published:** 2015-09-16

**Authors:** Jian Xu, Ying Ding, Vincent Malic

**Affiliations:** 1 School of Information Management, Sun Yat-sen University, Guangzhou, China; 2 Department of Information and Library Science, Indiana University, Bloomington, Indiana, United States of America; 3 University Library, Tongji University, Shanghai, China; Katholieke Universiteit Leuven, BELGIUM

## Abstract

Transdisciplinary collaboration is the key for innovation. An evaluation mechanism is necessary to ensure that academic credit for this costly process can be allocated fairly among coauthors. This paper proposes a set of quantitative measures (e.g., t_credit and t_index) to reflect authors’ transdisciplinary contributions to publications. These measures are based on paper-topic probability distributions and author-topic probability distributions. We conduct an empirical analysis of the information retrieval domain which demonstrates that these measures effectively improve the results of harmonic_credit and h_index measures by taking into account the transdisciplinary contributions of authors. The definitions of t_credit and t_index provide a fair and effective way for research organizations to assign credit to authors of transdisciplinary publications.

## Introduction

In 2009, transdisciplinary collaboration among materials scientists, immunologists, and bioengineers lead to the development of implantable, synthetic polymer matrices that dramatically enhanced host immunity and induced tumor regression in mice with established melanoma tumors [1]. This study is but one demonstration of the exciting results that can be achieved when scientists cross the established boundaries of their respective fields and synthesize an innovative approach to an intractable research problem. In general, the research questions being addressed by researchers throughout academia are growing in complexity, which in turn has resulted in a burgeoning interest in transdisciplinary research and the promotion of collaboration between and among various industries, organizations, and academic disciplines [2]. While various rationales may exist for embarking on interdisciplinary endeavors [3], a prevailing, problem-oriented view considers transdisciplinarity as a means to deal with complex research questions that cannot be addressed from the perspective of a single discipline. Such problems require people from different disciplines to work together, and to find a solution through the exchange of ideas, theoretical approaches, and best practices [4]. Since transdisciplinary research and collaboration can provide substantial benefits to scientists, practitioners, and policy makers [5], both public and private funding agencies have concrete incentives to provide significant support and funding to efforts which enable transformative collaboration across domains [6]. The growing interest in the nature of transdisciplinary research is evident in the growing number of studies on transdisciplinarity over certain domains. For example, analyses of transdisciplinary collaboration have been conducted in cognitive science [7], library and information science [8], the social sciences [9], and health sciences [10].

Transdisciplinarity yields specific benefits. As an approach to research, it can handle high levels of complexity, tap otherwise isolated sources of local knowledge, foster transformative thinking, and enhance legitimacy. At the same time, transdisciplinary research can be costly, because collaboration across disciplines and stakeholders is resource-intensive, error-prone, and time consuming [11]. In light of both the costs and the benefits, we consider it important to develop a means to evaluate and facilitate transdisciplinary collaboration. However, most current author credit-assignment schemas do not take transdisciplinary collaboration into account. Previous studies focused on credit-assignment schemas among coauthors of a publication based on author rank, number of coauthors, coauthor role (e.g., first author, corresponding author), or number of citations [12].

To address this problem, we proposed t_credit and t_index as measures for integrating transdisciplinary contribution weight, harmonic_credit value, and citation count. The operating assumption of these measures is that researchers who contribute to transdisciplinary studies should receive additional credit due to the nature of those studies. The t_credit and t_index measures also take into account the number of coauthors per paper and the nature of a given author’s contribution to a paper to remove the possibility of inflationary and equalizing biases. The number of citations received is also considered as a proxy for the quality of the publication. These indicators are calculated as follows: first, the topic distributions of coauthors and publications are identified by applying the LDA algorithm; second, KL divergence between author-topic probability distribution and paper-topic probability distribution is calculated as Author Transdisciplinary Weights (ATW); and lastly, the ATW are integrated with harmonic_credit assignment schema and number of citations to form the t_credit and t_index indicators.

According to the empirical analysis in the information retrieval domain, the results showed that the t_credit, which integrates ATW and number of citations with harmonic_credit assignment schema, can accurately assign transdisciplinary credit to a given author and thus improve the prevailing methods of credit-assignment by incorporating the added-value of transdisciplinary research. Use of the t_index indicator creates a more effective ranking of authors transdisciplinary contributions than h_index rankings. The coefficient of ATW and harmonic_credit assignment schema dramatically influences the final value of t_index of authors, which is important when evaluating an author’s overall transdisciplinary contribution. The h_index measure, in its current form, is incapable of accounting for the growth and contributions of transdisciplinary research.

The rest of the paper is organized as follows. Section 2 reviews current research on the evaluation of transdisciplinary collaboration, the identification of coauthor expertise, and methods for assigning credit to coauthors. Section 3 describes the method to calculate the ATW and original credit, and provides definitions of the t_credit and t_index indicators. Section 4 evaluates these measures in the field of information retrieval by comparing them with analogous rankings produced by credit-assignment schemas and h_index measures. In section 5 we discuss the value of the proposed measures for organizations and institutions interested in measuring transdisciplinary contributions to scientific research.

## Literature Review

### Evaluation of Transdisciplinary Collaboration

With the growing interest in promoting transdisciplinary research and training, evaluation of scientific processes and outcomes associated with transdisciplinary research has become vitally important, as government agencies and private foundations invest increasing amounts of resources in the formation of transdisciplinary research centers and teams [13–14]. In the meantime, many evaluations of transdisciplinary collaboration reveal its time-consuming nature, insufficient appreciation or recognition, competing institutional demands, loss of autonomy in decision making, frustration due to lack of progress, and interpersonal conflict impeding participation in such efforts. Stokols et al. [13] developed a conceptual and programmatic framework for evaluating collaborative research and the public-policy outcomes of transdisciplinary science. They claimed that since many universities give high priority to individualized academic achievements when distributing merit and conducting promotion reviews, and simultaneously offer few incentives to engage in transdisciplinary collaboration, junior investigators are cautious about pursuing transdisciplinary projects. They are also deterred from such activities because transdisciplinary pursuits tend to be intrinsically more time-consuming than their intradisciplinary counterparts. Stokols [15] examined three types of collaboration: first, collaboration among scholars representing different disciplines; second, collaboration between academics and community practitioners representing professional and lay perspectives; and third, collaboration among community organizations across local, state, national, and international boundaries. Each type of transdisciplinary collaboration comes with its own unique set of contextual circumstances that can facilitate or hinder research. Stokols held that transdisciplinary scientific collaborations are labor-intensive and often evoke tensions and conflicts among participants (e.g., stemming from their different disciplinary world views, interpersonal styles, and departmental affiliations) that must be confronted and resolved if the team as a whole is to achieve its collaborative goals. Kessel and Rosenfield [16] discussed research programs that have successfully traversed discipline boundaries in a sustained fashion, and considered facilitating and constraining factors that have emerged from the analysis of this process. They concluded that researchers have more reasons to against collaboration, which include: sharing credit affects promotion, tenure, publications, and funding; journals discourage multiple authors; peer review is rendered difficult; and transdisciplinary research must be framed narrowly. Pohl [17] indicated that sustaining transdisciplinary collaboration requires that members’ incentives to remain involved exceed the personal costs incurred through their participation. One efficient incentive is to promote the value of collaboration through promotion and tenure decisions [16].

There is limited research on the quantitative aspects of transdisciplinary research. Bote et al. [18] analyzed the benefits of international collaboration in terms of scientific impact, and research involving larger numbers of countries tended to have greater impact. Leydesdorff and Shin [19] improved the fractional counting of citations in ranking multidisciplinary research units (e.g., universities) by normalizing the differences among fields of science in terms of differences in citing behavior. Wagner et al. [20] summarized the indicators that may be used for quantitatively identifying and assessing the output of interdisciplinary research. These indicators include co-authorship, co-invention, collaboration, references, citations, and co-citations. So far, there are no quantitative measures for evaluating the transdisciplinary factors for each individual author in coauthored publications. In this paper, a quantitative measure of author transdisciplinary factors was proposed to reflect authors’ transdisciplinary contributions in their publications.

### Coauthor Expertise

It is crucial to identify the research expertise of each author in coauthored publications when assigning weights to individual contributions to transdisciplinary research. This is not a trivial task, particularly when examining a large set of coauthored papers. Previous studies adopt two methods—a keyword method and a topic-modeling method—to characterize the expertise of each author.

Chua and Yang [21] identified author-topic areas based on the department, division, faculty, center, or school cited as an author’s institutional affiliation. By using this method, the authors’ smallest available sub-organizational units were captured and coded into categories. Chang and Huang [22] applied a similar method to identify coauthors’ disciplines according to the institutional affiliation listed in the articles. These kinds of approaches provide a consistent and reproducible way of characterizing an author’s area of expertise. Since coauthor affiliation information is usually limited, only a general area of expertise can be inferred by this method. Another method [23] assigned disciplinarity on the basis of Web of Science (WoS) subject categories. They admit that the disadvantage of this method is that it is more coarse-grained than assigning disciplinarity at the article level.

Topic modeling methods such as Latent Dirichlet Allocation (LDA) [24] have been widely used to extract latent topics from documents. Author-topic models are used to describe both the research interests and the area of expertise of a given author simultaneously. Tang, Jin and Zhang [25] proposed the author-conference-topic (ACT) model, which calculates the probability of a topic for a given author, the probability of a word for a given topic, and the probability of a conference for a given topic. Topic modeling has been applied broadly to analyze patterns of scholarly communication [26–28]. To annotate the expertise of authors in a global collaboration network, He, Ding, Tang, Reguramalingam and Bollen [29] adopted the ACT model to approximate authors’ areas of expertise. In this paper, we extract authors’ expertise from their publications using the topic model proposed by Tang et al. [25], and Kullback-Leibler divergences are calculated to quantify the transdisciplinary contributions of each author.

### Coauthor Credit

The proportion of multi-authored scientific papers has increased significantly over the past several years [30] and this growth, in turn, has brought about a lively discussion regarding ways individual contributions to collaborations can be fairly evaluated. Assigning credit to multiple authors remains a problematic task, as practices vary between different research domains, and there does not exist a universally accepted approach to allocating credit for multi-authored papers [31].

Routinely authorship credit is allocated either by issuing full publication credit to each coauthor or by dividing one credit equally among all coauthors [32]. These methods, though common, may be susceptible to equalizing and inflationary counting bias [33]. Many other reasonable credit-assignment schemas have been developed which allocate credit according to the number of coauthors, their ranks, or both. In our previous study [12], 15 author credit-assignment schemas were divided into three general categories (i.e., linear, curve, and other) according to their coauthor credit-distribution patterns. The distribution of linear credit-assignment schemas is represented by a straight line, with different slopes for different schemas. In this schema, the difference between two credit values assigned to adjacent authors is always a constant value. Representative schemas of linear credit-assignment model are fractional counting [34] and proportional counting [35]. The distribution of curve-type credit-assignment schemas is represented by a curve, where the ratio between two credit values assigned to adjacent authors is either a constant value or a dependent value, depending on author rank. Typical curve-distribution schemas include geometric counting [36] and the harmonic_credit assignment schema [33]. Other types of credit-assignment schemas have distributions that are not as orderly as those of either linear- or curve-type schemas. Most other schemas are combinations of multiple schemas that apply under different circumstances or are based on author ranks, with credit reinforcement for the first and corresponding authors. Representative schemas of this type are the “sequence determines credit” (SDC) method [37], and the combined credit allocation (CCA) method [38].

In this paper, we adopt the harmonic_credit assignment schema [33] as the measure of credit-assignment factor, and integrate it with ATW and number of citations to form the t_credit and t_index measures. The harmonic_credit assignment schema can simultaneously remove both inflationary and equalizing bias by allocating publication credit according to authorship rank and the number of coauthors.

## Methods

### Data Collection

Information retrieval was chosen as the test field because it is an intrinsically transdisciplinary field, one that brings together scholars from information science and computer science in particular, and their techniques and tools have been applied in many other domains including the natural sciences, social sciences, and humanities.

Papers and their cited references were harvested from Web of Science (WoS) for the period ranging from 1956 to 2014. Search strategies were based on the following terms (including plurals and variants), which were determined by checking Library of Congress subject headings and consulting several domain experts: INFORMATION RETRIEVAL, INFORMATION STORAGE and RETRIEVAL, QUERY PROCESSING, DOCUMENT RETRIEVAL, DATA RETRIEVAL, IMAGE RETRIEVAL, TEXT RETRIEVAL, CONTENT BASED RETRIEVAL, CONTENT-BASED RETRIEVAL, DATABASE QUERY, DATABASE QUERIES, QUERY LANGUAGE, QUERY LANGUAGES, and RELEVANCE FEEDBACK. Under these constrains, 20,359 documents were found.

To disambiguate author names, we employed a simple 2-step procedure based on author name and affiliation [39]. First, we extracted each author’s full name and affiliation from the AF and C1 fields of the original downloaded WoS data. Second, the author’s full name and affiliation were used as a unique identifier capable of distinguishing one author from another. For example, we allocated the same unique identification code to two author instances, if they both had the same full name ‘Lalmas, Mounia’ and belong to the same affiliation ‘Yahoo Res Barcelona, Barcelona 08018, Spain’. In another case, if they have the same full name ‘Lalmas, Mounia’ and one belongs to the affiliation ‘Yahoo Res Barcelona, Barcelona 08018, Spain’ while another belongs to the affiliation ‘Univ Glasgow, Glasgow, Lanark, Scotland’, they are given two different identification codes on the assumption that they represent two different authors who happen to have the same name. This author disambiguation method is simple and is effective at distinguishing authors in a majority of cases. However, it has the drawback of classifying an author who changes affiliation as multiple people. Incomplete affiliation data in the WoS data constituted a further limitation. Of the 20,359 downloaded records, 17,847 contained affiliation information, which meant that for those authors who have no affiliation information, the author name was the only basis for disambiguation. After applying this method we identified 44,770 distinct authors in the dataset.

### Measure of Author Transdisciplinary Weight

The ACT model was used to generate a publication-topic distribution and an author-topic distribution. Then author transdisciplinary weight was calculated based on these distributions in the following steps:

#### Step 1: Use LDA to obtain paper-topic probability (PTP) distribution vectors

Topic modeling has been widely used to extract latent topics from text corpora [24, 40–41]. We uesd the ACT model proposed by Tang et al. [25] to obtain a paper-topic probability distribution for the information retrieval dataset. The inputs of the model are the titles, authors, and publication venues (e.g., journals, conferences) of the selected texts. The model then outputs a probability distribution of papers over topics.

#### Step 2: Calculate author-topic probability distribution

Vectors of author-topic probability distribution (ATP) were calculated by using all paper-topic probability distribution vectors that correspond to an author. At this stage we did not take into account the position of the author in the list of authors or the number of coauthors, since these factors are present in the harmonic_credit schema, which in turn is integrated into the final calculation of the t_credit and t_index measures. Thus, if a paper has multiple authors, the same PTP is used for the calculation of the ATP for each of the paper’s authors. For each author:

$$ATP=\frac{1}{n}\sum_{i=1}^{n}PTP_{i},$$ (1)

where *PTP*_*i*_ is the i-th paper-topic probability distribution vector of the author, and n is the total number of papers that the author has published.

#### Step 3: Calculate Kullback-Leibler divergence

We then calculated KL divergence between author-topic probability distribution and paper-topic probability distribution. The higher KL divergence of an author, the stronger transdisciplinary contribution he or she made. On the other hand, a lower KL divergence means that the topics of papers that the author has published are more similar to the topics of current paper than those of other authors:

$$D_{KL\_r}(ATP_{r}||PTP)=\sum_{x=1}^{D}ATP_{r}(\text{x})\text{log}\frac{ATP_{r}(\text{x})}{PTP(\text{x})}\text{ (1}\leq \text{r}\leq \text{N),}$$ (2)

where is the KL divergence value between the r-th author’s ATP and PTP, D is the dimension of ATP and PTP vector, and N is the total number of coauthors of a paper. By definition.

Note that we cannot just take as author transdisciplinary weight because the scale of the KL divergence value is comparatively small (ranging from 0 to 0.35) and it will dramatically shrink the original integration result. We therefore take the logarithm of this value to produce a range that can be appropriately integrated with other measures of author credit. We call the logarithmic transformation of the Author Transdisciplinary Weight (ATW):

$$ATW=\frac{-1}{{\text{log}}_{e}(\left|D_{KL}{}_{\_r}\right|+\alpha )}$$ (3)

where ATW is the Author Transdisciplinary Weight (ATW) ranging approximately from 0 to 1 (from 0.11 to 0.95 in our dataset). Parameter *α* ensures has a value is greater than zero. In this study, we set *α* = 0.0001. The limitation of ATW is that the authors’ research domains are derived from their published papers. Thus the transdisciplinary contributions of those who have published more papers can be derived better than those who have published fewer.

### Measure of Original Author Credit

Current author credit-assignment schemas routinely rely on two counting methods: inflated counting, where full authorship credit is issued repeatedly to all coauthors (also known as total, normal, or standard counting), and fractional counting, where one credit is divided equally among all coauthors. Fractional counting corrects for the inflationary bias generated by the multiple counting of multi-authored publications, but both counting methods generate equalizing bias by dividing credit uniformly among all coauthors, irrespective of their actual contribution. We adopt the harmonic_credit assignment schema to calculate the original author credit, since this schema can simultaneously removes both inflationary and equalizing bias by allocating publication credit according to authorship rank and the number of coauthors [33].

The distribution of harmonic_credit assignment schemas can be represented as a curve, where the ratio between two credit values assigned to adjacent authors is either a constant or a dependent value related to author rank.

**Fig 1:**
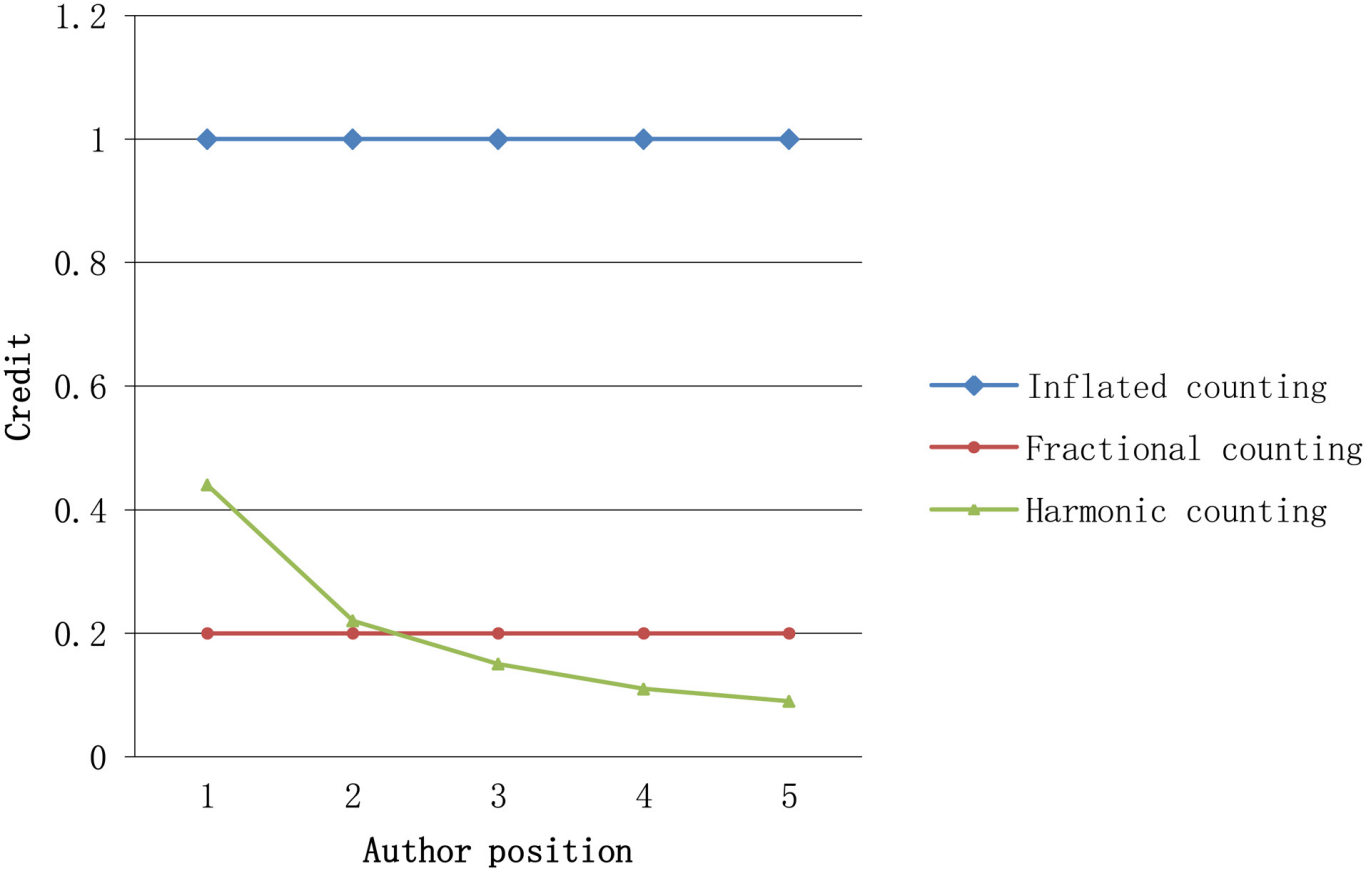
The representative credit-assignment schemas’ distribution.

Fig 1 shows the distribution for each representative credit schema using a paper with five coauthors. The distributions of inflated counting and fractional counting are horizontal lines, as they assign each coauthor an equal portion of the credit (in this case 1 and 0.2). Compared to the inflated counting schema and the fractional counting schema, harmonic counting appears more intuitive as the difference between lower-ranked coauthors is small while major coauthors receive most of the credit.

If we let N be the number of coauthors of a publication, and r be the rank of an author, then harmonic_credit assignment schema can be described by the formula:

$$harmonic\_credit_{r}=\frac{1/r}{\left[1+1/2+\cdot \cdot \cdot +1/N\right]},\text{ 1}\leq r\leq N,$$ (4)

where harmonic_credit is the credit value of the rth-ranked author. The ratio of credits between the rth author and the r+1th author in this schema is. In this paper, we assume that sequence of authors should reflect the declining importance of their contribution. However, if some situations (e.g., corresponding author listed in the last position, alphabetical order of authors, etc.) needed to be taken into account, harmonic_credit schema can be replaced by other proper credit allocation schemas [12].

### Measures of Author Transdisciplinary Contribution

Measuring an author’s transdisciplinary contribution is a challenging task. It needs to integrate the three basic aspects of transdisciplinarity evaluation: the credit to be assigned, the citation count of the paper, and the extent to which an author’s contribution can be considered transdisciplinary. The distribution of original credit should be a reasonable allocation among coauthors depending on their position in the listing of authors and number of coauthors. The citation number serves as an approximative indicator of a publication’s quality. We measure transdisciplinarity by evaluating the topic divergence between the topic of a given paper and the topics with which an author has been associated. Incompletely considering any of the above factors may result in bias in the estimation of transdisciplinary contribution.

For example, from Table 1 we can see that in group 1, Losee RM and Lalmas M have similar mean citations (11.05 and 10.75) and mean ATW values (0.40 and 0.42). But their mean credit values are different (0.90 and 0.50), since Losee published most of his papers as the first author (20 out of 21) while Lalmas only published a minority of her articles as the first author (8 out of 20), which means that Losee’s contributions should be weighted more than Lalmas in regards to author precedence. Indicators that lack a weighted credit schema would suffer from an equalizing bias, even if these indicators incorporated both paper citation count and ATW.

**Table 1:** Examples of evaluating the author transdisciplinary contribution.

Group	Authorname	Paper number	First author	Mean citation	Mean ATW	Mean harmonic_credit	Harmonic_credit sum	Harmonic_credit rank	H_index
1	LOSEE, RM	21	20	11.05	0.40	0.90	19.00	3	11
1	LALMAS, M	20	8	10.75	0.42	0.50	10.02	20	6
2	CRESTANI, F	31	17	13.52	0.40	0.53	16.41	4	11
2	CROFT, WB	27	12	58.19	0.37	0.54	14.67	6	18
3	JACSO, P	12	12	17.25	0.20	1.00	12.00	11	6
3	RADECKI, T	11	11	20.36	0.44	1.00	11.00	14	7

In group 2, both Crestani F and Croft WB have similar mean credit value (0.53 and 0.54), and they have similar mean ATW value (0.40 and 0.37). But their mean citations show a remarkable difference (13.52 and 58.19), which means that, on average, papers published by Croft have more impact than those of Crestani. Therefore, citation count is also very important when evaluating transdisciplinary contribution, since it reflects variance in publication quality.

The indicator of transdisciplinarity is an essential measure to quantify an author’s transdisciplinarity contribution. We see this when comparing Jacso P and Radecki T in group 3. These two researchers have the same mean credit (1.0) and similar mean citations (17.25 and 20.36), but their mean ATW values are obviously different (0.20 and 0.44), which means Radecki has had greater involvement in transdisciplinary efforts than Jacso while still maintained the quality of publications.

To better evaluate the overall performance of an author’s transdisciplinary contributions, we need to integrate these three indicators: harmonic_credit, citation count, and ATW. The tricky part of integration is how to integrate these indicators in an efficient way while they have different ranges of values. Our two integration proposals, t_credit and t_index, are outlined below.

#### T_credit

T_credit improves the harmonic_credit schema via the integration of citation count and ATW. We calculate the t_credit of an author for a given paper with the following formula:

$$t\_credit=harmonic\_credit\times \text{ATW}\times {\text{log}}_{10}citation$$ (5)

In order to evaluate the effectiveness of this means of integrating all three indicators, we also created two variations of the t_credit schemas based on the integration of harmonic_credit separately with citation count (formula 6) and ATW (formula 7). In section 4, formula (5–7) is calculated based on the information retrieval dataset, and the comparison between them justifies that the integration of both 3 aspects is necessary.

$$t\_credit_{HC}=harmonic\_credit\times {\text{log}}_{10}citation$$ (6)

$$t\_credit_{HA}=harmonic\_credit\times \text{ATW}$$ (7)

The purpose of logarithm of citation number is that the original citation number can cause major impact on the final result. While normalizing the citation number into range 0 to 1 seems irrational since number of citations between different publications can vary dramatically, the logarithm of the citation number can shrink the range of citation number to cater to the integration needs.

Then each author’s total credit is calculated as follows:

$$t\_credit_{sum}=\sum_{i=1}^{n}t\_credit_{i}$$ (8)

where n is the number of the author’s publications.

We consider t_credit_sum_ to constitute an improved author-credit schema in that it takes both paper quality and transdisciplinarity into account. As we see in formula (8), an author’s total t_credit is the sum of the product of each publication’s harmonic_credit, ATW value, and the logarithm of the paper’s citation count. An author’s t_credit_sum_ serves as a more complete measure of an author’s contribution than the index calculated by harmonic_credit alone.

#### T_index

Since scientometrics researchers first introduced the h_index as a measure of scientific impact [42], much research has been devoted to improving its performance and accuracy [33, 43–46]. We have yet to see an improved version of the h_index that takes transdisciplinarity and a weighted distribution of author credit into account. Since the h_index lacks these factors, we believe that in some respects the h_index is an incomplete measure. Two authors with equal h-indices (e.g., the same number of papers with the same number of citations) have not made equal contributions if one author is always the first author while the other is always the third author. In a similar vein, two authors with the same h-indices and the same author positions have nonetheless made qualitatively different contributions if one has been involved in more transdisciplinary efforts while the other has not.

Here we propose the t_citation measure, which integrates ATW, harmonic_credit, and citation count. The t_citation measure is used in the calculation of the t_index measure. We define the t_citation measure as follows:

t_citation=harmonic_credit×ATW×citation (9)

For comparison purposes, we define two other t_citation measures as citation count integrated separately with either harmonic_credit or ATW.

$$t\_citation_{HC}=harmonic\_credit\times citation$$ (10)

$$t\_citation_{AC}=\text{ATW}\times citation$$ (11)

Note that here we do not take the logarithm of the citation count. This is because in this formulation we only take harmonic_credit and ATW as parameters of the citation count. By multiplying the citation count by harmonic_credit, citations can be allocated to each coauthor according to their position in the byline and number of coauthors; by multiplying citation count by the ATW, those who contribute more in the transdisciplinary research will be weighted more.

Inspired by the definition of h_index, we define the t_index based on the new definition of citation:

*A scientist has t_index if t of his/her N papers have at least t t_citations each*, *and the other (N − t) papers have no more than t_citations each*.

T_index_HC_ and t_index_AC_ are alternate versions of t_index that are formulated using t_citation_HC_ or t_citation_AC_, respectively. T_index_HC_ appears to be equivalent to the harmonic h_index measure proposed by Hagen [33]. Hagen developed the harmonic h_index to remove inflationary and equalizing biases from the original h_index while retaining the essential simplicity, transparency and intended fairness of the original h_index. In this paper we insist that transdisciplinarity should also be taken into account when evaluating authors given the importance of transdisciplinarity for collaborative science and innovation.

## Results and Discussion

### T_credit

We applied t_credit schema to the information retrieval publications (see Table 2) to demonstrate the value of t_credit.

**Table 2:** Difference in ranks of the 20 top-ranked authors based on the harmonic_credit schema and t_credit schema.

Author	No. of pub.	F pub.(%)	Citation mean	ATW mean	Harmonic credit mean	Harmonic credit sum	Harmonic credit rank	T_credit_HCsum_	T_credit_HCsum_ rank	T_credit_HAsum_	T_credit_HAsum_ rank	T_credit_sum_	T_credit_sum_rank	Rank change
SALTON, G	30	80	118.57	0.34	0.70	20.85	1	31.02	1	7.04	3	10.28	2	-1
SPINK, A	36	75	44.56	0.42	0.57	20.41	2	25.09	2	8.55	1	10.61	1	1
LOSEE, RM	21	95	11.05	0.40	0.90	19.00	3	17.52	5	7.53	2	6.84	4	-1
CRESTANI, F	31	55	13.52	0.40	0.53	16.41	4	13.27	11	6.69	4	5.29	8	-4
SAVOY, J	17	88	12.41	0.38	0.87	14.85	5	14.09	8	5.59	6	5.14	9	-4
CROFT, WB	27	44	58.19	0.37	0.54	14.67	6	18.40	4	5.43	7	6.81	5	1
CHEN, HC	35	49	34.54	0.44	0.39	13.59	7	19.41	3	5.81	5	8.34	3	4
BORGMAN, CL	17	94	35.59	0.28	0.78	13.26	8	13.31	10	3.98	18	4.47	15	-7
EGGHE, L	15	100	12.33	0.37	0.82	12.33	9	10.28	18	4.51	12	4.08	19	-10
JACSO, P	12	100	17.25	0.20	1.00	12.00	10	10.57	17	2.43	62	1.96	82	-72
BLAIR, DC	13	100	35.92	0.42	0.92	12.00	11	14.11	7	5.04	8	5.92	6	5
FUHR, N	19	63	23.47	0.30	0.60	11.36	12	12.22	13	3.73	21	4.45	16	-4
RADECKI, T	11	100	20.36	0.44	1.00	11.00	13	11.93	14	4.83	9	5.03	10	3
GARFIELD, E	11	100	72.64	0.43	1.00	11.00	13	14.86	6	4.69	10	4.81	14	-1
WILBUR, WJ	14	79	12.93	0.40	0.77	10.85	15	8.25	32	4.33	16	3.35	27	-12
JARVELIN, K	35	20	13.29	0.44	0.30	10.65	16	9.24	22	4.65	11	3.97	22	-6
SMEATON, AF	19	53	8.05	0.40	0.56	10.57	17	5.97	67	4.39	14	2.54	48	-31
SPARCK-JONES, K	15	80	9.93	0.35	0.70	10.52	18	6.18	60	3.50	27	2.28	61	-43
LALMAS, M	20	40	10.75	0.42	0.50	10.02	19	5.46	83	4.16	17	2.30	60	-41
LIBKIN, L	19	42	9.63	0.17	0.50	9.56	20	6.36	57	1.73	142	1.11	256	-235

Note: No. of pub.Number of publications; F pub.(%) Papers as first author; Rank change Rank-order change of harmonic_credit_sum_ vs. t_credit_sum_; in Rank change column, zero indicates that rank order does not change, a positive number indicates that rank order increases, and a negative number indicates a decrease.

We use the examples from Table 1 here to demonstrate how t_credit works. In Table 2, Losee RM and Lalmas M have similar mean citation values and mean ATW values. The difference lies in their mean credit. Harmonic_credit accounts for this difference, and the values of the two authors in t_credit_sum_, t_credit_HCsum_ and t_credit_HAsum_ are consistent with their values in the harmonic_credit_sum_.

In another example in Table 2, both Crestani F and Croft WB have similar mean harmonic_credit values and mean ATW values, but the difference between their mean citation counts is remarkable. Since the harmonic_credit and t_credit_HA_ do not account for citation count, these authors have similar harmonic_credit_sum_ and t_credit_HAsum_. A higher citation count, however, indicates that the quality of Croft’s publications is higher than that of Crestani’s, all other factors being equal. As we can see in Table 2, harmonic_credit_sum_ and t_credit_HAsum_ fail to express the real credit difference of these two authors while t_credit_HCsum_ and t_credit_sum_ can effectively distinguish them. Comparing to the harmonic_credit rank, the Crestani’s t_credit_sum_ rank has dropped from 4 to 8, while the Croft’s t_credit_sum_ rank has raisen from 6 to 5.

ATW is another important factor which harmonic_credit did not take into account when generally evaluates an author’s transdisciplinarity contribution. Though Jacso P and Radecki T have made similar contributions garnering similar numbers of citations, Radecki has participated in more transdisciplinary pursuits. The values and ranks of harmonic_credit_sum_ and t_credit_HCsum_ can hardly distinguish the differences in their transdisciplinary contributions, while the t_credit_HAsum_ and t_credit_sum_ properly reflects this difference. Under the t_credit_sum_ ranking, Radecki has risen from 13 to 10, while Jacso drops from rank 10 to 82.

In sum, harmonic_credit_sum_ is a suitable measure for credit allocation among coauthors only when factoring their position in the coauthor list and the number of coauthors; t_credit_HCsum_ is better than harmonic_credit_sum_ when the citation count needs to be considered; and t_credit_HAsum_ is better than harmonic_credit_sum_ when taking transdisciplinarity into account. When the need arises to consider all three of these factors, t_credit_sum_ is the best choice.

The same rank counts of the 100 top-ranked authors ordered by harmonic_credit in different segments are listed in Table 3. The corresponding segmented cumulative counts are listed in Table 4.

**Table 3:** Segmented counts of the 100 top-ranked authors based on harmonic_credit.

Rank	Rank 1–20 (RecNum: 20)	Rank 21–40 (RecNum: 20)	Rank 41–60 (RecNum: 20)	Rank 61–80 (RecNum: 23)	Rank 80–100 (RecNum: 17)
t_credit_HCsum_	14(70%)	9(45%)	2(10%)	2(9%)	2(12%)
t_credit_HAsum_	16(80%)	11(55%)	9(45%)	7(30%)	2(12%)
t_credit_sum_	13(65%)	8(40%)	1(5%)	3(13%)	0(0%)

Note: The segments are not separated evenly because tied ranking cases are not uncommon in ranking lists. In each cell, the number means the counts of authors who have the same rank range in both ranking list of harmonic_credit and ranking list of t_credit_sum_, and the percentage in brackets means the percentage of the counts of authors who have the same range in both ranking list.

**Table 4:** Segmented cumulative counts of the 100 top-ranked authors based on harmonic_credit.

Rank	Rank 1–20 (RecNum:20)	Rank 1–40 (RecNum:40)	Rank 1–60 (RecNum:60)	Rank 1–80 (RecNum:83)	Rank 1–100 (RecNum: 100)
t_credit_HCsum_	14(70%)	28(70%)	42(70%)	59(71%)	70(70%)
t_credit_HAsum_	16(80%)	33(83%)	51(85%)	71(86%)	81(81%)
t_credit_sum_	13(65%)	25(63%)	42(70%)	57(69%)	67(67%)

Note: The segments are not separated evenly because tied ranking cases are not uncommon in ranking lists. In each cell, the number means the counts of authors who have the same rank range in both ranking list of harmonic_credit and ranking list of t_credit_sum_, and the percentage in brackets means the percentage of the counts of authors who have the same range in both ranking list.

In Table 3, we compare the rank differences between harmonic_credit, t_credit_HCsum_, t_credit_HAsum_, and t_credit_sum_ in different segmented ranges. We see, for example, that in the t_credit_sum_ ranking, 13 individuals have the same rank they were assigned in the harmonic_credit ranking in rank 1 to 20. When we compare lower rank segments, however, the similarities between the harmonic_credit ranking and the t_credit rankings decrease significantly. We interpret these differences as reflecting the fact that the intellectual giants of the information retrieval field will, for the most part, retain the highest ranks irrespective of the type of measure used, while the differences captured by the different indices become more evident as we move further down the ranks. Note that the largest difference is between harmonic_credit and t_credit_sum_ the measure which simultaneously accounts for author credit distribution, citation count, and ATW.

In Table 4, we compared the rank differences between harmonic_credit, t_credit_HCsum_, t_credit_HAsum_ and t_credit_sum_ in different segmented cumulative ranges. By counting the same rank in different segmented cumulative ranges, we can also find out that there exist differences between rank of harmonic_credit and other ranks and the most obvious difference lies between harmonic_credit and t_credit_sum_ (67(67%), in segment from 1 to 100), which is consistent with the result of Table 3.

The same-rank count differences between the harmonic_credit ranking and the t_credit rankings relfect the limitations of the harmonic_credit schema. During allocating credits to authors, harmonic_credit was only calculated based on the author position in the coauthor list and the number of coauthors, and it did not take into account the citation factor and ATW factor. However, t_credit_HC_ integrates the citation counts and therefore it can differentiate authors who have similar position, number of coauthors and number of papers, but citations of papers are different, which imply that their papers have different impact. For example, in Table 1 we can see that both Crestani F and Croft WB have similar mean harmonic_credit value, but the remarkable difference lies in their mean citations. If we adopt harmonic_credit here to evaluate above two authors, it is unfair to Crestani since his papers have more impact in general. T_credit_HC_ can deal with this situation since the citation factor is taken into account. ATW is another important factor which harmonic_credit did not take into account. For example, in Table 1, Jacso P has similar harmonic_credit mean value with Radecki T, but their difference in transdisciplinary contribution cannot be differentiate by only adopt harmonic_credit. By integrate the ATW factor with harmonic_credit, t_credit_HA_ can reflect the influence of transdisciplinary contribution. T_credit_sum_, accounts for both citation count and transdisciplinarity, and therefore can be considered a more adaptable measure than t_credit_HC_ and t_credit_HA_.

### T_index

**Fig 2:**
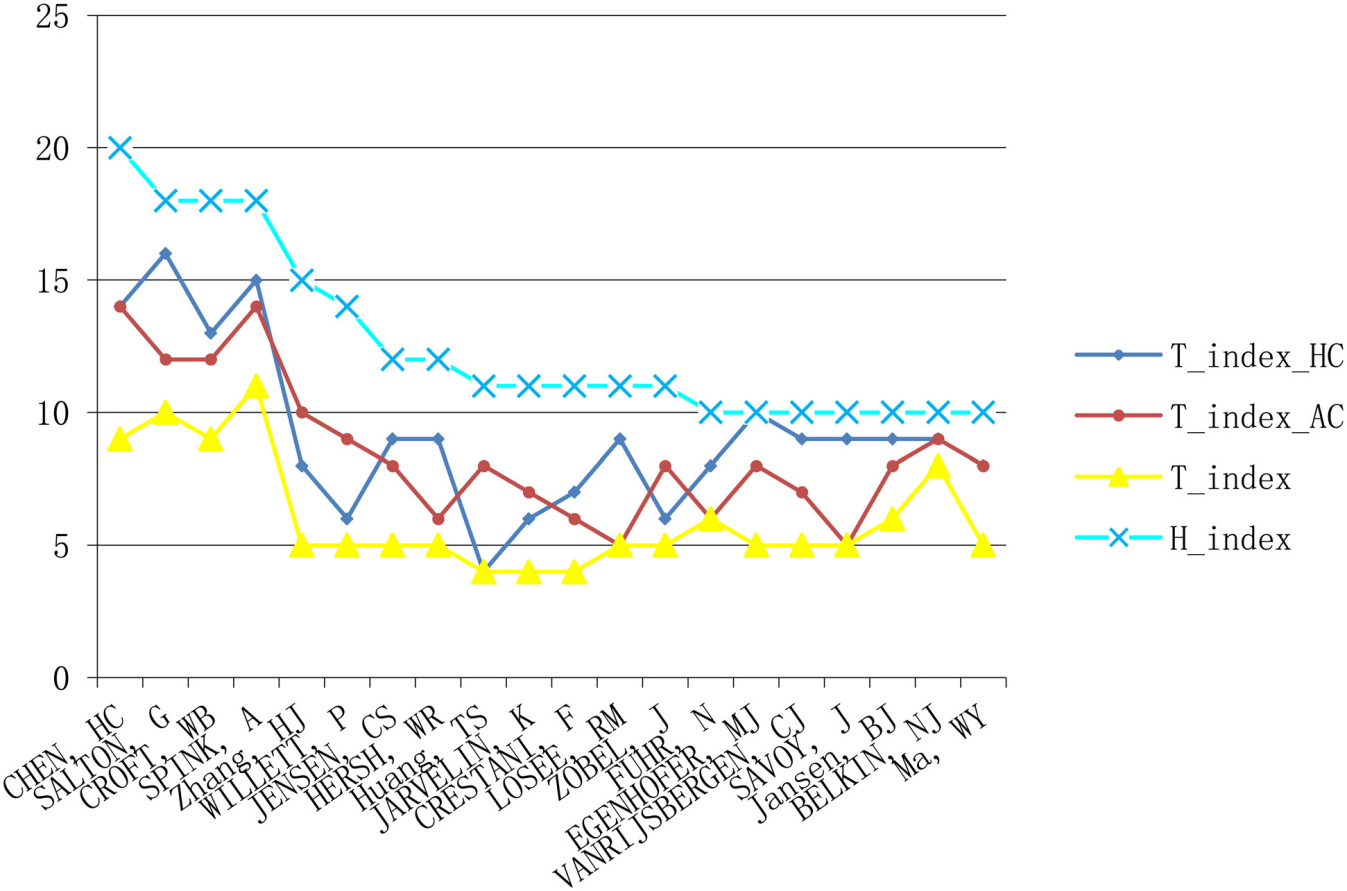
The comparison between values of t_index and h_index.

**Fig 3:**
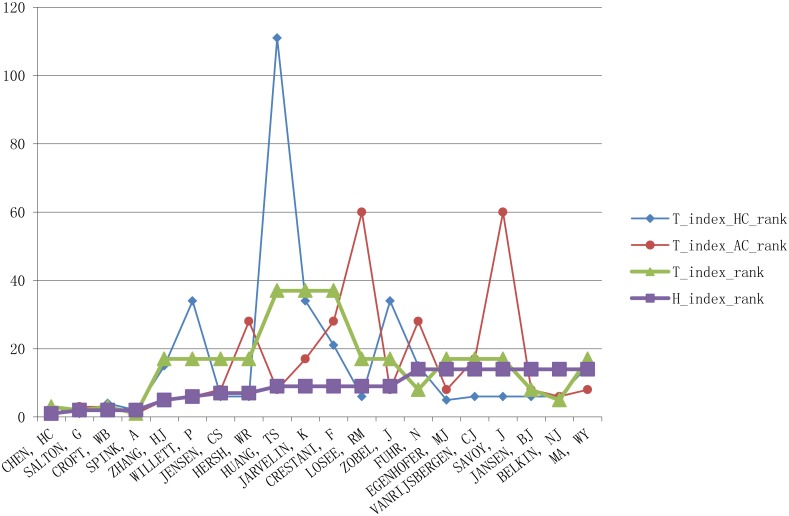
The comparision between ranks of t_index and h_index.

We calculated the h_index, t_index_HC_, t_index_AC_ and t_index values and ranks for each author in the information retrieval dataset. We compare the index values in Fig 2 and author ranks in Fig 3. As discussed above, we remove equalizing and inflationary bias by incorporating the harmonic_credit schema and account for transdisciplinarity through the integration of ATW.

Note in Fig 2 that the curve of h_index is always higher than other indexes, and the curve of t_index is lower than other indexes. The reason lies in that the value of ATW and harmonic_credit is always equal to or smaller than 1. By multiply with ATW or harmonic_credit or both of them, the value of t_citaiton_HC_, t_citation_AC_ and t_citation will equal to or be smaller than the number of original citations. Consequently, the value of t_index_HC_, t_index_AC_, and t_index which were calculated based on t_citation_HC_, t_citation_AC_, and t_citation will always be smaller than or at most equal to the value of h_index. We can see from Fig 2 that there exist obvious differences between these indices. For example, Croft WB and Spink A have the same h_index (18). But when harmonic_credit and ATW indicators are taken into account separately, Spink has larger values of t_index_HC_ (15) and t_index_AC_ (14) than Croft (13 and 12). Not surprisingly, since the t_index reflects the comprehensive effect of both the harmonic_credit and ATW indicators, the Spink’s t_index (11) is expectedly bigger than Crofts (9). In another example, Losee RM and Zobel J also have same h_index (11). But when harmonic_credit and ATW indicators are taken into account separately, Losee, however, receives a higher t_index_HC_ value while Zobel’s t_index_AC_ value is larger. The t_index measure balances the simultaneous impact of the harmonic_credit indicator and the ATW indicator, and therefore Losee and Zobel share the same t_index value (6). In general, the value of t_index is influenced by citation count, harmonic_credit, and ATW, and its value distribution in Fig 2 can be seen as a fitted curve of t_index_HC_ and t_index_AC_ values.

From Fig 3 we can see that the t_index, t_index_HC_, and t_index_AC_ rankings have different distributions from the h_index ranking. In general, the distribution of t_index ranking in Fig 3 can be seen as a fitted curve of the t_index_HC_ and t_index_AC_ rankings and those who contribute more to transdisciplinary research and have higher harmonic_credit scores will have higher t_index ranks. For example, Jensen CS is ranked 7th by h_index, but drops to 17th on the t_index ranking. Further investigation shows that his average harmonic_credit value is 0.33, which is lower than the average value of top 20 authors (0.49). His average ATW value is 0.33, which is also lower than the average value of the top 20 authors (0.39). Interaction between harmonic_credit and transdisciplinary factors causes the rank to drop. Since the h_index does not integrate harmonic_credit or factor transdisciplinarity into its formulation, it is insensitive to these drops. Another example can further explain the advantage of t_index. Belkin NJ rises from 14 on the h_index ranking to the 5th on the t_index ranking. Again the interaction between credit and transdisciplinary factors lead to the changes, but this time they increase his rank. According to our investigation, Belkin’s average harmonic_credit value is 0.57, which is higher than average value of the top 20 authors (0.49). His average ATW value is 0.44, which is higher than the average value of top authors (0.39).

The same rank counts of the 106 top-ranked authors ordered by h_index in different segments are listed in Table 5. The corresponding segmented cumulative counts are listed in Table 6.

**Table 5:** Segmented counts of the 106 top-ranked authors based on h_index.

Rank	Rank 1–20 (RecNum: 20)	Rank 21–37 (RecNum: 17)	Rank 38–65 (RecNum: 28)	Rank 66–106 (RecNum: 41)
t_index_HC_	15(75%)	7(41%)	5(18%)	0(0%)
t_index_AC_	15(75%)	4(24%)	11(39%)	0(0%)
t_index	17(85%)	3(18%)	0(0%)	14(34%)

Note: The segments are not separated evenly because tied ranking cases are not uncommon in ranking lists. In each cell, the number means the counts of authors who have the same rank range in both ranking list of h_index and ranking list of t_index, and the percentage in brackets means the percentage of the counts of authors who have the same range in both ranking list.

**Table 6:** Segmented cumulative counts of the 106 top-ranked authors based on h_index.

Rank	Rank 1–20 (RecNum:20)	Rank 1–37 (RecNum:37)	Rank 1–65 (RecNum:65)	Rank 1–106 (RecNum:106)
t_index_HC_	15(75%)	31(84%)	52(80%)	81(76%)
t_index_AC_	15(75%)	29(78%)	58(89%)	87(82%)
t_index	17(85%)	30(81%)	39(60%)	86(81%)

Note: The segments are not separated evenly because tied ranking cases are not uncommon in ranking lists. In each cell, the number means the counts of authors who have the same rank range in both ranking list of h_index and ranking list of t_index, and the percentage in brackets means the percentage of the counts of authors who have the same range in both ranking list.

In Table 5, we compare the rank differences of the top 106 authors with respect to h_index, t_index_HC_, t_index_AC_ and t_index in different segmented ranges. Although by multiplying harmonic_credit or ATW or both of them, the values of t_index_HC_, t_index_AC_ and t_index will be less than the corresponding value of h_index in general, and this will cause the decrease of discrimination between authors, we can still clearly see that there exist obvious differences between rank of h_index and other ranks, and the differences increase while the segmented ranges increase from rank 1–20 to rank 66–106 in general.

In Table 6, we compare rank differences between h_index, t_index_HC_, t_index_AC_, and t_index in cumulative segmented ranges. The increasing difference with the increasing rank number observed in Table 5 is again visible. From Tables 5 and 6 we can see that there are significant differences between h_index and t_index, which are accounted for by the integration of harmonic_credit and ATW values. The integration of both harmonic_credit and ATW with citation count provides a more comprehensive schema, which better reflects the extent of an authors’s contribution when transdisciplinarity and coauthorship are taken into account.

## Conclusions

With the swift advance in science and technology, structured or unstructured data are outpouring into our daily life, ranging from data collected from satellites, data sensored by personal wearable devices, ever-expanding data digitized from personal genome sequencing and electronic medical records, to user-generated data distributed via Facebook, Twitter, and other social medias. The complexity of data is beyond what a single specialist can handle. The context of data is highly tied with the domains and specific problems in sciences, social sciences, and humanities. Making sense of data is not just as simple as clicking few buttons on some software. It requires a transdicplinary team to work together to better understand, store, analyze, and interpret data. The analytic process is recursive and collaborative that heavily involves domain experts to re-collect, re-analyze, and re-interpret data in order to a big picture. The greatest challenging of making sense of data is notechnical, rather the right mix of a transdisciplinary team with disparate backgrounds which includes scientist who understands the domain, data scientists who know how to run computing efficiently and effectively, and managers who have strong leadership and right vision [47]. Transdisciplinary collaboration without walls and divisions is essential for the success of "big science".

To encourage transdisciplinary collaboration, the academic evaluation system should be adapted to promote cross-disciplinary research. For the long decades, transdisciplinary contributions have been ignored, and were never integrated into any major evaluative metrics (e.g., citations, publications, h_index). Previously, it is understandable that the scientific research can be handled by a single team or even an individual, collaboration was never necessay and mainstream. But now scientific innovation is often triggered by transformative research, and working around the boundaries is the most exicting part of science but evaluating people on the boundaries is hard. To fill the gap, this paper proposed the t_credit and t_index measures, which are capable of accurately quantifying an author's transdisciplinary contribution to research papers.

The major contribution of this paper is to propose a measure of author transdisciplinary contribution and define the t_credit and t_index indicators which integrate transdisciplinary (i.e., ATW), author credit (i.e., harmonic_credit) and quality (i.e., number of citations). We begin by taking paper-topic probability distribution vectors using Latent Dirichlet Allocation. Then author-topic probability distribution vectors are calculated by using all paper-topic probability distribution vectors corresponding to a given author. ATW is then calculated as the KL divergence between author-topic and paper-topic probability distributions. Finally, we integrate ATW with harmonic_credit and citation counts in two different fashion to obtain t_credit and t_index values. Empirical data analysis in the field of information retrieval demonstrates clearly that credit ranks and h_index ranks change noticeably when transdisciplinarity is taken into account. Further analyses show that interaction of ATW, harmonic_credit and citation counts can effectively improve the results of harmonic_credit or h_index when measuring the transdisciplinary contribution of authors.

Transdisciplinary collaboration can provide substantial benefits to scientists, practitioners, and policy makers [3] and many scholars predict that the future of research is increasingly interdisciplinary [6]. Approporiate evaluation mechanisms are needed to ensure this costly process can be managed fairly and maintained sustainably. The limitation of proposed methods is that the authors’ research domains are derived from their published papers. Therefore, these methods can better reflect the transdisciplinary contributions of senior researchers, who have published more papers in their domains, than those of junior researchers, who have published fewer papers in their domains.

The t_credit and t_index provide quantitative measures for research organizations to measure their researchers’ transdisciplinary contributions in transdisciplinary research fields. Looking forward, we plan to apply these measures to evaluate researchers’ transdisciplinary contributions in different stages of their research careers to identify how transdisciplinary contributions influence a scientist’s career trajectory. An author’s contribution is tightly connected with the entities they are working on. We want to narrow the transdisciplinary contribution down to the entity level to see how an author’s transdisciplinary contribution can connect siloed entity clusters. We hope to enrich our measurements of transdisciplinary contributions by considering the mutual influence of entities, papers, journals, and co-authors. We also want to modify and apply our measures to understand and capture the evolution of disciplines and transdisciplines.

## Supporting Information

S1 File The 100 top-ranked authors based on harmonic_credit. Supporting data for Tables 1, 3 and 4.
(CSV)

S2 File The 106 top-ranked authors based on h_index. Supporting data for Tables 5 and 6.
(CSV)
